# New Insights into the Role of Oxidative Stress in the Development of Diabetes Mellitus and Its Complications

**DOI:** 10.1155/2023/9824864

**Published:** 2023-07-26

**Authors:** Julia Matzenbacher dos Santos, Qing Zhong, Sandra A. Benite-Ribeiro, Thiago Gomes Heck

**Affiliations:** ^1^Health Promotion and Development Department, School of Nursing, University of Pittsburgh, Pittsburgh, USA; ^2^Department of Biomedical Sciences, Rocky Vista University, Ivins, USA; ^3^Programa de Pós-graduação em Ciências Aplicadas à Saúde, Universidade Federal de Jataí, Jataí, Brazil; ^4^Postgraduate Program in Integral Health Care, Regional University of Northwestern Rio Grande do Sul State, Ijuı´, Rio Grande do Sul State, Brazil

The special issue addresses new insights into the role of oxidative stress in the development of diabetes mellitus and its complications. Diabetes is a pandemic that has reached alarming proportions; it is estimated that the global prevalence in adults (between 20- and 79-year-olds) will reach 12.2%, which is over 783 million people, by 2045 [1]. Poor glycemic control, the common threat for individuals with all types of diabetes, impacts micro- and macrovasculature homeostasis [2]. As a result, several complications, such as cardiovascular events, retinopathy, neuropathy, and nephropathy, are induced in the population with diabetes and are the leading cause of mortality and morbidity [3–5]. Together, diabetes and its complications generate a significant economic burden, and its global health-related expenditures were estimated to reach almost a trillion USD in 2021 [1].

A large number of cutting-edge studies have established that oxidative stress is one of the central pieces of the development of diabetes and its complications, as highlighted in the previous special issue published in this journal [6]. The big challenge in the field is the fact that molecules involved in oxidative stress response, such as reactive oxygen species (ROS), cannot be eliminated completely from the human body, since they control vital processes in the human body such as the immunological defense, mitochondrial biogenesis, and antioxidant system. The establishment of the “ideal” balance between generation and scavenging of ROS is the target of controlling diabetes development and the complications related to this disease. Therefore, scientists worldwide have focused on characterizing ROS-related sources and behaviors, their associated pathways, and scavenging components in diabetes.

Novel insights linking oxidative stress in the field of diabetes and its complications have been well addressed in the present thematic issue. This special issue includes seven research articles (two reviews of literature and five original articles) focusing on the role of ROS in the development of diabetes and its associated diseases. The guest editors are thrilled to present a collection of innovative, original research and review scientific reports as follows.

Several researchers have shown that regular exercise inhibits and delays the development of type 2 diabetes [7]; however, most of the studies in the diabetes field were carried out analyzing the effect of aerobic (mainly running) or resistance exercise. In the review article “The Effects of Tai Chi Exercise for Patients with Type 2 Diabetes Mellitus: An Overview of Systematic Reviews and Meta-Analyses,” the impact of regular practice of tai chi, which is a series of calm physical exercises and stretches' movement in individuals with type 2 diabetes, was evaluated [8]. The authors concluded that Tai Chi is beneficial and safe for individuals with type 2 diabetes; however, due to few experimental research on the topic, professionals in the area should approach this conclusion with caution.

In the original research, “Serum Uric Acid Levels Are Related to Diabetic Peripheral Neuropathy, Especially for Motor Conduction Velocity of Tibial Nerve in Type 2 Diabetes Mellitus Patients” and determine the role of serum uric acid in the neuropathy of patients with type 2 diabetes mellitus [9]. Analyzing 106 type 2 diabetes patients with and without diabetic neuropathy, the authors suggest that serum uric acid levels could impact the function of the tibial nerve motor fiber independent from the control of glycated hemoglobin. Altogether, the authors concluded that subnormal serum uric acid is a risk factor for neuropathy of patients with type 2 diabetes mellitus.

Another original research is about a traditional Chinese medicine prescription (GuaLouQuMaiWan) that has been described as an alternative treatment for type 2 diabetes mellitus (T2DM) with positive results [10]. With a mix of compounds, with unclear small molecular components, the study by Feng et al. used network pharmacology and transcriptomics to reveal its mechanism in treating diabetes. After gene expression analysis, up- and downregulated genes were investigated, related to insulin secretion and proinflammatory profile. In an interestingly health-to-disease process, from no disease to glucose intolerance to type 2 diabetes profile, seventeen genes were highlighted by the authors. The progression until established diabetes involves several abnormalities in *β* cells, including a decrease in the number of pancreatic *β* cells and their function during the secretion and synthesis of insulin. Thus, they started to test whether someone within the fifty compounds of traditional medicine met the criteria by interaction of target proteins. In this way, this study found associations between genes related to inflammatory targets such as cytokines, nuclear transcription factors, growth factors, and energy balance. Thus, the results of this study [10] help to explain the molecular pathways involved in diabetes pathogenesis and provide new strategies for treatment, such as specifically reducing the degree of inflammation in pancreatic islets. Since pharmacological and nonpharmacological interventions for diabetes have been discussed based on inflammatory, oxidative, and proteostasis pathways [11], it is possible to propose that the compound could restore the number of *β* cells and islet function by connecting *in silico*, *in vitro*, animal models, and clinical data pieces of evidence, to possibly becoming effective in patients with failed insulin control, based on anti-inflammatory and insulin resistance properties.

Traditional Chinese medicine is not the only alternative intervention beneficial to patients with diabetes, multiple vitamins' supplementation including vitamins B, C, and E decreases the development of diabetic retinopathy. The original research “The Amelioration of Detrimental Biochemical Anomalies by Supplementing B, C, and E Vitamins in Subjects with Type 2 Diabetes Mellitus May Reduce the Rate of Development of Diabetic Retinopathy” was a prospective placebo control trial. In this study, Pramanik et al. followed 185 patients with T2DM who received vitamin B, vitamin C, and vitamin E together with antidiabetic medication and 175 patients with T2DM who were treated with only antihyperglycemic drugs for five years. The group with vitamin supplementation had a slower rate of the development of diabetic retinopathy and reduced ROS markers [12]. It is concluded that vitamin supplementation decreases the development of diabetic microvascular complications via their antioxidant properties.

Myrrh resin, a natural substance obtained from the bark of the *Commiphora myrrha*, has been tested as a therapeutical approach against several diseases. In the original research “Pharmacological Studies on the Antidiabetic, Antioxidant, and Antimicrobial Efficacies of *Commiphora myrrha* Resin in Streptozotocin-Induced Diabetes in Rats: A Preclinical Study” [13], the authors tested the hypotheses that aqueous extract of *Commiphora myrrha* resin has an antioxidant, antimicrobial, and antidiabetic effect for type one diabetes (T1D). Oleo-gum resins of *Commiphora myrrha* were collected from a wild tree growing in Wadi Noeman at Makkah, Saudi Arabia, and aqueous resin oleum were extracted as described by the authors [13]. Streptozotocin-induced diabetes in rats were treated *Commiphora myrrha* powder (0.5 mL of 0.5 g/kg body weight) dissolved in water for 30 days. The results of the study successfully supported the hypothesis, which opens a window of opportunity for testing myrrh resin administration to control blood glucose of T1D in clinical trials.

Chronic inflammation impairs wound healing in individuals with diabetes. Controlling oxidative stress is one of the therapeutics approaches to improve diabetic-induced cutaneous wounds that precede diabetic foot ulcers. Using cell culture of alternatively activated macrophage (M2 phenotypes) that is responsible to inflammation and tissue regeneration, the authors of the original article “ceAF Ameliorates Diabetic Wound Healing by Alleviating Inflammation and Oxidative Stress via TLR4/NF-*κ*B and Nrf2 Pathways” tested the hypotheses that chick early amniotic fluids (ceAF) ameliorate inflammation and wound healing [14]. The results of the study indicate that ceAF downregulates inflammatory response by the regulation of TLR4/NF-*κ*B and Nrf2 signaling pathways in M2 macrophage and that could improve diabetic wound healing.

The global incidence and the prevalence of type 1 diabetes mellitus (T1DM) are increasing rapidly. Vitamin D, which underlies calcium and phosphorus metabolism, also has an immunomodulatory role. Therefore, researchers have been testing the hypothesis that decreased levels of vitamin D underlie chronic infections, specific types of cancer, and autoimmune rheumatic diseases [14]. Scientific reports on the relationship between vitamin D deficiency and the development of T1DM are discussed in the review article “Progress in the Relationship between Vitamin D Deficiency and the Incidence of Type 1 Diabetes Mellitus in Children” [15]. Together, studies indicated that vitamin D could protect pancreatic *β* cells from immune attack by regulating T cell response via reducing oxidative stress. However, according to the reports analyzed in the present literature review, there is no consensus regarding the protective effect of supplementation of vitamin D on *β* cell function in T1DM. Therefore, large-scale epidemiological studies are needed to evaluate the role of vitamin D in the development of T1DM.

The guest editors expect this special issue to be of huge interest to scientists and clinicians working in the field of diabetes and/or oxidative stress, especially those focusing their work on alternative therapeutical approaches against the development of diabetes and its complication. We hope that investigators worldwide continuously make additional advances in expanding the knowledge in the field of diabetes and its complication.
